# Length and Geometric Patterns of the Greater Palatine Canal Observed in Cone Beam Computed Tomography

**DOI:** 10.1155/2010/292753

**Published:** 2010-08-15

**Authors:** Karen Howard-Swirzinski, Paul C. Edwards, Tarnjit S. Saini, Neil S. Norton

**Affiliations:** ^1^Department of Oral Biology, Creighton University, Omaha, NE 68178, USA; ^2^Department of Periodontics and Oral Medicine, University of Michigan School of Dentistry, Ann Arbor, MI 48109, USA; ^3^US Army DENTAC, Brooke Army Medical Center, Fort Sam Houston, San Antonio, TX 78234, USA

## Abstract

The greater palatine canal is an important anatomical structure that is often utilized as a pathway for infiltration of local anesthesia to affect sensation and hemostasis. Increased awareness of the length and anatomic variation in the anatomy of this structure is important when performing surgical procedures in this area (e.g., placement of osseointegrated dental implants). We examined the anatomy of the greater palatine canal using data obtained from CBCT scans of 500 subjects. Both right and left canals were viewed (*N* = 1000) in coronal and sagittal planes, and their paths and lengths determined. The average length of the greater palatine canal was 29 mm (±3 mm), with a range from 22 to 40 mm. Coronally, the most common anatomic pattern consisted of the canal traveling inferior-laterally for a distance then directly inferior for the remainder (43.3%). In the sagittal view, the canal traveled most frequently at an anterior-inferior angle (92.9%).

## 1. Introduction

The anatomy of the greater palatine canal is of interest to dentists, oral maxillofacial surgeons, and otolaryngologists performing procedures in this area (e.g., administration of local anesthesia, dental implant placement, orthognathic Le Fort I osteotomies, and sinonasal surgeries) [1–6]. It houses the descending palatine artery (a branch of the third division of the maxillary artery) and greater and lesser palatine nerves (branches of the maxillary division of the trigeminal nerve) and their posterior inferior lateral nasal branches [2]. The trigeminal nerve provides sensory innervation to all of the maxillary and mandibular teeth and surrounding tissues. The trigeminal nerve splits into three branches in the middle cranial fossa, which exit through separate foramina. The maxillary division of the trigeminal (V2) exits the skull through the foramen rotundum where it transverses high in the pterygopalatine fossa. It enters the floor of the orbit and bone again anteriorly through the inferior orbital fissure in the posterior maxilla and travels towards the face. The maxillary division innervates all maxillary teeth, maxillary palatal and gingival tissue, skin of the midface, the nasal cavity, and sinuses [7]. The nerve of the pterygoid canal also enters the pterygopalatine fossa from the posterior, usually slightly inferior to the foramen rotundum, and transmits the nerve of the pterygoid canal. The greater palatine canal travels inferiorly from the pterygopalatine fossa, housing the greater palatine and lesser palatine nerves, which diverge to enter the hard palate at respective foramina [7]. 

Blocking sensation of the maxillary nerve in the pterygopalatine fossa by administering a maxillary division block achieves anesthesia to all of the above mentioned structures. A common technique to achieve a maxillary division block is the greater palatine canal approach in which a needle is inserted through the greater palatine foramen and advancing the needle until it is in the inferior portion of the pterygopalatine fossa, where anesthetic is deposited. Infiltration of local anesthetic into the greater palatine canal can also be employed to obtain vasoconstriction during endoscopic sinus surgery (ESS) [1]. In this procedure, the needle is advanced to the limit of the greater palatine canal, but not into the fossa, to avoid the potential complication of arterial puncture [1]. Further advancement of the anesthetic syringe to reach the infraorbital nerve, located deep in the pterygopalatine fossa, is required when regional maxillary anesthesia is desired [1, 4, 5]. Therefore, knowing the anatomy and average lengths of the greater palatine canal is important when employing these techniques.

The walls of the greater palatine canal are formed anteriorly by the infratemporal surface of the maxilla, posteriorly by the pterygoid process of the sphenoid, and medially by the perpendicular plate of the palatine [7]. The maxillary sinus is located anterior, and the nasal cavity and concha medial and the pterygoid plates posterior to the greater palatine canal. The anatomy of these structures undoubtedly affects the anatomy of the greater palatine canal due to their proximal relationships. When performing surgical procedures in this area, preservation of the descending palatine artery and palatine nerves is essential to avoid excessive bleeding and to maintain nerve supply to the maxilla [8]. In other cases, regional nerve block may be unsuccessful if excessive resistance is met when injecting local anesthesia into the greater palatine canal, presumably the result of anatomic variation. 

The purpose of this investigation was to determine the average length of the greater palatine canal and identify the most common anatomic pathways of this structure using cone beam computed tomography (CBCT) data obtained from patients at a dental school setting.

## 2. Methods

CBCT data obtained from 500 patient scans were reviewed. The CBCT scans were obtained between August 2005 and April 2007 at Creighton University School of Dentistry for a variety of dental indications. Scans were performed at 0.3 mm voxels. Canals were viewed and analyzed in both sagittal and coronal planes. Xoran technologies (Imaging Sciences International) i-CAT workstation program was used to visualize the data and to record canal path and length. 

**Figure 1:**
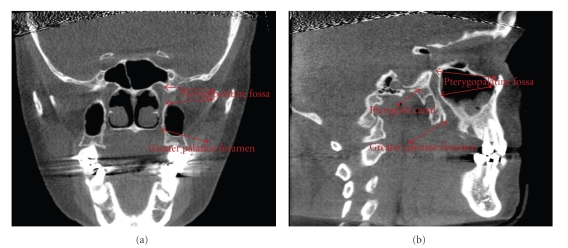
The images above demonstrate the appearance of anatomical structures on CBCT images described above; coronal view (a) and sagittal view (b). From the coronal view, the pterygopalatine fossa and greater palatine canal can be seen lateral to the nasal cavity. The pterygopalatine fossa begins below the middle cranial fossa and meets the greater palatine canal below which extends to enter the hard palate at the greater palatine foramen. In the sagittal view, the pterygopalatine fossa and greater palatine canal can be seen again, posterior to the maxillary sinus. The pterygoid canal is visible here, entering the pterygopalatine fossa from the posterior. The midpoint of the pterygoid canal was determined in this plane for each canal and used as the superior point of measurement.

The length and anatomic paths traveled by both the right and left greater palatine canals (*N* = 1000) were determined. While both the foramen rotundum and pterygoid canal enter the pterygopalatine fossa from the posterior aspect, their locations are variable [9]. For this study, the pterygoid canal was selected as the superior limit instead of the foramen rotundum due to its ease of identification in relation to the greater palatine canal. Thus, the length of the greater palatine canal was defined as the bony portion of the greater palatine canal measured from the center of the pterygoid canal, as the center point of the pterygopalatine fossa, to the greater palatine foramen on the inferior surface of the hard palate. Soft tissue depth was not included. The pterygoid canal was marked in a superior-inferior direction with the use of the program's line coordinates so its vertical location was known while navigating through plane slices. The greater palatine canal was then measured from the marked vertical level to the apparent opening at the greater palatine foramen on the hard palate of both coronal and sagittal sections. In the sagittal plane, the inferior limit of the greater palatine canal was measured to the posterior wall of the great palatine foramen and in the coronal plane to the inferior surface of the horizontal hard palate for standardization due to variance in the foramen shape. Length of the canal was measured in millimeters using the Xoran software, following the most straight-line path through the center of the canal. The path of the greater palatine canal was recorded as the description of the descending length tracing lines in the canals. A compass was used on the CBCT images to record deviation from vertical. Length and path trends were analyzed for averages with standard deviations. The major anatomical landmarks are shown in Figure 1.

## 3. Results

Of the 500 subjects, 265 (53%) were female and 235 (47%) were male ranging in age from 18–73. The average length of the greater palatine canal was 29 mm (±3 mm), ranging from 22 to 40 mm. 

**Figure 2:**
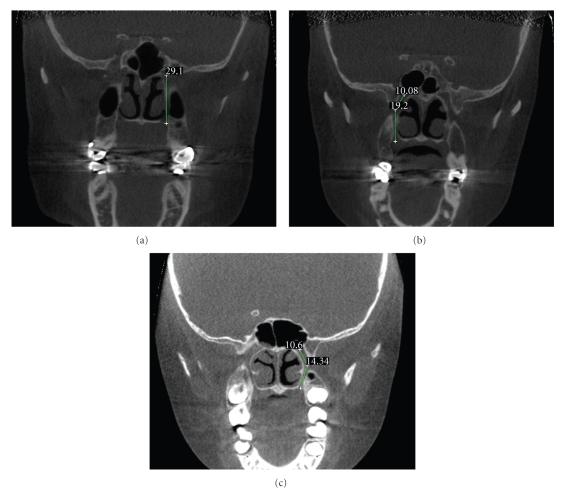
The coronal images above, showing unilaterally traced canals of three different subjects, were selected as examples of the most common canal pathways observed in the medial-lateral plane. The images also demonstrate how the canal paths and lengths were determined; the most straight-line path in the center of the canal, superiorly from the midpoint of the pterygoid canal entrance into the pterygopalatine fossa to the inferior surface of the horizontal hard palate. In some cases, bilateral canals could be traced in the same sagittal slice if both appeared patent (c), but in most cases only one patent canal was visible in a single slice and navigation anterior or posterior was required to see the other. Most common canal pathways demonstrated in the images are (a) canal travels directly inferior from fossa, (b) canal travels inferior-lateral for a distance then directly inferior for the remainder, and (c) canal travels inferior-lateral for a distance then inferior-medial for the remainder.

**Figure 3:**
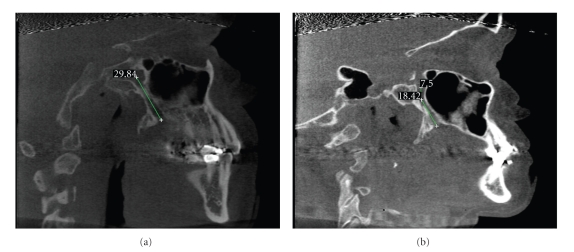
The unilateral sagittal images above, from two different subjects, were selected as examples of the most common canal pathways in the anterior-posterior plane. Most common sagittal canal pathways as demonstrated in the images are (a) canal travels anterior-inferior and (b) canal travels directly inferior for a distance then anterior-inferior for the remainder.

The directional pathways observed in the coronal plane are summarized in Figure 2. Three pathways were consistently observed: (1) the greater palatine canal travels directly inferior from the pterygopalatine fossa (Figure 2(a)), (2) the greater palatine canal travels inferior-lateral for a distance then changes direction to pass directly inferior for the remainder of the canal (Figure 2(b)), and (3) the greater palatine canal travels inferior-lateral for a distance then changes direction to pass inferior-medial for the remainder of the canal (Figure 2(c)). 

The directional pathways observed in the sagittal plane are summarized in Figure 3. In this plane, two pathways were observed: (1) the greater palatine canal travels in an anterior-inferior direction from the pterygopalatine fossa (Figure 3(a)) and (2) the greater palatine canal travels directly inferior for a distance and then changes direction to pass anterior-inferior for the remainder of the canal (Figure 3(b)).

The incidences of the directional pathways are summarized in Table 1 and the average angles and directional distances are summarized in Table 2. In the coronal plane, the most common pathway observed was the greater palatine canal traveling inferior-lateral for a distance then changing direction to pass directly inferior for the remainder of the canal (Figure 2(b)). This occurred in 43.3% of the total canals. In these cases, the average angle from the vertical was 28 (±6) degrees and occurred for 8 (±2) mm before traveling inferiorly. The next most frequent pathway was observed when the greater palatine canal traveled directly inferior from the pterygopalatine fossa (Figure 2(a)). This pathway occurred in 39.5% of the canals. The third pathway observed in the coronal plane was when the greater palatine canal traveled inferior-lateral for a distance then changed direction to pass inferior-medial for the remainder of the canal (Figure 2(c)). This occurred in 16% of the population. In these cases, the average angle from the vertical was 25 (±7) degrees and occurred for 10 (±3) mm before traveling medially at an average angle of 11 (±5) degrees from the vertical for the remainder of the canal. 

In the sagittal plane, the most common pathway was the greater palatine canal travels in an anterior-inferior direction from the pterygopalatine fossa (Figure 3(a)), which was observed 92.9% and an average angle of 27 (±6) degrees. In 6.5% of the canals, the greater palatine canal traveled directly inferior for a distance and then changed direction to pass anterior-inferior for the remainder of the canal (Figure 3(b)). In these cases, after traveling directly inferior from the pterygopalatine fossa for 9 (±4) mm, the angle from the vertical was 33 (±6) degrees and occurred for 8 mm before traveling inferiorly.

## 4. Discussion

The use of the greater palatine canal as a route for injection of local anesthetic has many advantages. In studies by Wong and Sved [4] and Lepere [5], they note that the maxillary nerve block would be advantageous for palatal surgery, periodontal surgery involving maxillary teeth, Caldwell-Luc procedure, quadrant restorative dentistry of the maxilla, multiple extractions, or a diagnostic aid due to local infection. Buddor summarizes use of the block in general anesthetics for awake intubation [10]. Additionally, it is indicated for hemostasis and anesthesia in endoscopic sinus surgery, septorhinoplasty, and posterior epistaxis [1, 11]. According to Wong and Sved [4], the absolute contraindication for use of the maxillary nerve block technique is when there is palatal swelling located around the greater palatine foramen. Like any maxillary nerve block, several complication are possible, including intravascular injection, nasal bleeding, diplopia, neural injury, anesthetic failure (due to incorrect angulation, insufficient needle penetration, inability to locate the greater palatine foramen, or intravascular injection), and insufficient anesthesia [3, 5, 12]. 

Since several of the procedures for which the palatine block may be indicated are generally of a more complex clinical nature (e.g., dental implant placement), it is not unreasonable that the clinician may have already have CBCT data obtained prior to the procedure. In this situation, the clinician may wish to analyze the anatomy of the greater palatine canals in a manner similar to that employed in this study to determine the potential likelihood of encountering complications. However, in the absence of such pretreatment CBCT data, a number of conclusions derived from this study are of benefit to the clinician. Previously, most data had been collected from human skulls. Malamed and Trieger thoroughly examined 204 skulls and observed that the optimal angle for needle penetration was 45.88 degrees and in over 97% of the skulls a probe could be passed from the greater palatine foramen into the pterygopalatine fossa without difficulty [6]. With CBCT we were able to observe the exact pathway of the greater palatine canal. The anterior posterior path of the canal appeared to be relatively consistent, with 92.9% of canals traveling at a straight anterio-inferior angle. The medial lateral anatomy was more varied, with the most common anatomy being a straight inferior path (encountered 39.5%). However, depending on the variation in this pathway, the difficulty in passing a needle from the greater palatine foramen to the pterygopalatine fossa can be understood.

The recommended length of insertion of the anesthetic needle into the greater palatine canal has been suggested to be anywhere from 25 mm (for hemostasis in sinus surgery) to 32–39 mm (for maxillary anesthesia) [1, 4, 5, 13]. Unusually long canals could lead to lack of anesthesia. Conversely, unusually short canals could have a higher occurrence of complications if standard needle advancement lengths are utilized. Therefore, knowledge of the average lengths of the canal is beneficial. Several previous studies have examined the length of the palatine. A cadaveric study from Thailand found the combined length of the greater palatine canal and pterygopalatine fossa to be 29.7 ± 4.2 mm, including 6.7 ± 2.3 mm of soft tissue [3]. Computed tomography studies have shown the length of the greater palatine canal to range from 27 to 40 mm, depending on the definition of the superior limit, excluding the soft tissue [13]. The results of this study fell within previously established averages and ranges. 

## Figures and Tables

**Table 1:** Incidence of pathways of greater palatine canal. The table below summarizes the frequency of canal pathways observed in both the medial-lateral and anterior-posterior planes, unilaterally (out of each 500 right and 500 left), bilateral symmetry (out of 500 pairs), and overall incidence (out of 1000 right and left canals).

Figure	Pathway	Right canal	Left canal	Bilaterally symmetrical	Overall incidence
Medial-Lateral (Coronal) Direction					

Figure 2(a)	Canal travels directly inferior from fossa	45% (223)	34% (172)	22% (112)	39.5%
Figure 2(b)	Canal travels inferior-lateral for a distance then directly inferior for the remainder	39% (193)	48% (240)	23% (114)	43.3%
Figure 2(c)	Canal travels inferior-lateral for a distance then inferior-medial for the remainder	15% (77)	17% (83)	6% (31)	16%
	Other	1% (7)	1% (5)	0	1.2%

Anterior-Posterior (Sagittal) Direction					

	Canal travels anterior-inferior	91% (456)	94.5% (473)	88% (441)	92.9%
Figure 3(a)	Canal travels directly inferior for a distance then anterior-inferior for the remainder	8% (40)	5% (25)	2% (10)	6.5%
Figure 3(b)	Other	1% (4)	0.5% (2)	0.02% (1)	.06%

**Table 2:** Average angles and directional distances of observed canal pathways. The table below summarizes the average angle and distance traveled in each component when a canal traveled of the vertical in each of the major canal pathways in both planes. The straight inferior trend viewed in the coronal plane is not included because canals following this pathway followed a direct vertical path.

Figure	Pathway	Directional distance	Right canal	Left canal
Medial-Lateral (Coronal) Direction				

Figure 2(b)	Canal travels inferior-lateral for a distance then directly inferior for the remainder	Inferior-lateral angle (degrees)	28 (±6)	28 (±6)
		Inferior-lateral distance (mm)	8 (±2)	8 (±4)

Figure 2(c)	Canal travels inferior-lateral for a distance then inferior-medial for the remainder	Inferior-lateral angle (degrees)	25 (±7)	23 (±3)
		Inferior-lateral distance (mm)	10 (±3)	11 (±4)
		Inferior-medial angle (degrees)	11 (±5)	11 (±4)

Anterior-Posterior (Sagittal) Direction				

Figure 3(a)	Canal travels anterior-inferior	Anterior-inferior angle (degrees)	27 (±6)	27 (±6)

Figure 3(b)	Canal travels directly inferior for a distance then anterior-inferior for the remainder	Directly inferior distance (mm)	9 (±4)	8 (±3)
		Anterior-inferior angle (degrees)	33 (±6)	33 (±6)
